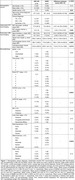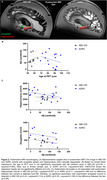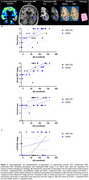# Correlating antemortem amyloid PET burden, postmortem MRI volumes, with neuropathological measures: Insights from Down syndrome and Alzheimer's disease cohorts

**DOI:** 10.1002/alz70856_103737

**Published:** 2025-12-26

**Authors:** Jr‐Jiun Liou, Tales Santini, Milos D. Ikonomovic, Victor L Villemagne, Ann D Cohen, Charles M Laymon, Davneet S Minhas, Weiquan Luo, Dana L Tudorascu, Benjamin L Handen, Oscar L Lopez, William H Yong, Mark Mapstone, Bradley T Christian, Sigan L Hartley, Adam Brickman, Julie C Price, H. Diana Rosas, Florence Lai, Shahid Zaman, Elizabeth Head, Julia K. Kofler, Tamer S Ibrahim

**Affiliations:** ^1^ University of Pittsburgh, Pittsburgh, PA, USA; ^2^ University of California, Irvine, Irvine, CA, USA; ^3^ University of Wisconsin‐Madison, Madison, WI, USA; ^4^ Columbia University, New York, NY, USA; ^5^ Massachusetts General Hospital, Harvard Medical School, Boston, MA, USA; ^6^ University of Cambridge, Cambridge, United Kingdom

## Abstract

**Background:**

In late‐onset AD, β‐amyloid (Aβ) deposition is associated with limbic‐predominant age‐related TDP‐43 encephalopathy neuropathologic change (LATE‐NC), beginning in the amygdala and spreading to the hippocampus and neocortex. The association in Down syndrome (DS) remains unexamined. People with DS are genetically predisposed to early AD, with Aβ accumulation occurring at younger ages. This study investigates the relationship between Aβ deposition and postmortem volumes or neuropathologic changes in DS and late‐onset AD.

**Methods:**

Demographic and clinical diagnoses—cognitively stable (CS), mild cognitive impairment (MCI), and dementia—were collected from the Alzheimer Biomarker Consortium – Down Syndrome (ABC‐DS) and Pittsburgh Alzheimer's Disease Research Center (ADRC). Results of Aβ PET scans with 11C‐Pittsburgh compound B and 18F‐florbetapir were expressed in centiloids. Amygdala and hippocampus were manually segmented from postmortem ex vivo 7‐tesla MRI. Histopathology included Thal phase, Braak NFT stage, CERAD score, LATE‐NC stage, hippocampal sclerosis, Lewy body stage, arteriolosclerosis, atherosclerosis, and cerebral amyloid angiopathy. T‐tests, linear regressions, and ANCOVA were performed to compare cohorts, examine associations between Aβ and MRI or neuropathology metrics, and compare regression slopes of two cohorts.

**Results:**

The ABC‐DS cohort showed similar Aβ PET burden but had a lower prevalence of ApoE4 carriers, younger age at clinical diagnosis and autopsy, shorter PET‐autopsy interval, smaller amygdala and hippocampal volumes, more advanced CERAD scores, and no atherosclerosis compared to ADRC cohort (Figure 1). In our existing datasets, the association between age at PET scan and Aβ burden did not reach statistical significance likely due to limited sample size. Antemortem Aβ burden was not associated with postmortem amygdala or hippocampal volumes in either group (Figure 2). Higher Aβ burden was associated with advanced LATE‐NC stage in ABC‐DS but not in ADRC. In ADRC, Aβ burden correlated with postmortem Aβ plaque, neurofibrillary tangles, and neuritic plaques, while no significant associations were found in ABC‐DS for Thal phase, Braak NFT stage, or CERAD score (Figure 3).

**Conclusions:**

In DS, antemortem Aβ PET burden is associated with advanced LATE‐NC stage but not with postmortem amygdala or hippocampal volumes. In ADRC cohort, Aβ burden correlates with postmortem amyloid plaque, neurofibrillary tangle, and neuritic plaque pathology.